# Serotonin, Inhibition, and Negative Mood

**DOI:** 10.1371/journal.pcbi.0040004

**Published:** 2008-02-01

**Authors:** Peter Dayan, Quentin J. M Huys

**Affiliations:** 1 Gatsby Computational Neuroscience Unit, University College London, London, United Kingdom; 2 Center for Theoretical Neuroscience, Columbia University, New York, New York, United States of America; University College London, United Kingdom

## Abstract

Pavlovian predictions of future aversive outcomes lead to behavioral inhibition, suppression, and withdrawal. There is considerable evidence for the involvement of serotonin in both the learning of these predictions and the inhibitory consequences that ensue, although less for a causal relationship between the two. In the context of a highly simplified model of chains of affectively charged thoughts, we interpret the combined effects of serotonin in terms of pruning a tree of possible decisions, (i.e., eliminating those choices that have low or negative expected outcomes). We show how a drop in behavioral inhibition, putatively resulting from an experimentally or psychiatrically influenced drop in serotonin, could result in unexpectedly large negative prediction errors and a significant aversive shift in reinforcement statistics. We suggest an interpretation of this finding that helps dissolve the apparent contradiction between the fact that inhibition of serotonin reuptake is the first-line treatment of depression, although serotonin itself is most strongly linked with aversive rather than appetitive outcomes and predictions.

## Author Summary

Serotonin is an evolutionarily ancient neuromodulator probably best known for its role in psychiatric disorders. However, that role has long appeared contradictory to its role in normal function, and indeed its various roles in normal affective behaviors have been hard to reconcile. Here, we model two predominant functions of normal serotonin function in a highly simplified reinforcement learning model and show how these may explain some of its complex roles in depression and anxiety.

## Introduction

Serotonin (5-hydroxytryptamine [5-HT]) is a neuromodulator that appears to play a critical role in a wealth of psychiatric conditions, including depression, anxiety, panic, and obsessive compulsions. However, despite the importance of serotonergic pharmacotherapies, notably selective serotonin reuptake inhibitors (SSRIs), the roles that serotonin plays in normal and abnormal function are still mysterious. We start from three particular findings. First, 5-HT is involved in the prediction of aversive events, possibly as a form of opponent [1–3] to dopamine [4–11]. Second, 5-HT is involved in behavioral inhibition [12–14], preventing or curtailing ongoing actions in light of predictions of aversive outcomes. The third finding is the collection of psychopharmacological data implicating 5-HT in animal models of depression and anxiety [15–17], together with the fact that depleting 5-HT (by dietary depletion of its precursor, tryptophan) in human subjects who have recovered from depression, can reinstate an acute, at times fulminant, re-experience of subjective symptoms of the disease, as assessed by various rating scales [18–21]. Furthermore, while SSRIs are used in the treatment of depression, genetically induced, constitutive decreases in the efficiency of 5-HT reuptake are a risk factor for depression [22–24]. These findings are hard to connect: the second fact seems orthogonal to the first and third, which are themselves in apparent contradiction. If 5-HT is really involved in predicting aversive outcomes, then *depleting* it should surely have positive rather than negative affective consequences.

We suggest that the missing link comes from considering the interactions between Pavlovian predictions and ongoing action selection. The interaction is seen in conditioned suppression [25], a standard workhorse test for aversive predictions. Animals are trained to emit appetitive instrumental actions (such as pressing a lever for reward), and to associate (by classical conditioning) a light with a shock. Presentation of the light during instrumental performance *reduces* the rate at which animals emit those responses. Neither the theoretical nor the neurobiological status of this interaction is completely resolved, though there is some evidence of the involvement of 5-HT in the nucleus accumbens in its realization [26–28].

Here, we treat a subset of the inhibitory processes associated with Gray's behavioral inhibition system (BIS) [7,13,29,30] in terms of what might be called a preparatory Pavlovian response. Consummatory Pavlovian responses are (evolutionarily) pre-programmed reactions to the presence of affectively significant outcomes such as food, water, or threats. Preparatory Pavlovian responses are similarly pre-programmed responses to predictions of those outcomes. Even though the predictions are learned, the responses are not, and may therefore be behaviorally inappropriate in certain circumstances [31,32]. For our purposes, and as long noted by Deakin and Graeff [7], the most important preparatory Pavlovian response to a prediction of a (sufficiently distant) threat [30] is inhibition, in the form of withdrawal or disengagement. This explicitly links the first two findings discussed above, as the inhibition is directly associated with aversive predictions.

To explore the consequences of reflexive, direct inhibition of action for learning in affective settings, together with the repercussions when 5-HT is compromised, we built a highly simplified model that sought to isolate these effects from more general learning effects. More specifically, we built a model of trains of thoughts. In our treatment, we considered thoughts as actions that lead from one belief state to the next. Trains of thought gained value through their connections with a group of terminal states that were preassigned either positive or negative affective values. 5-HT directly inhibited chains of thought predicted to lead toward negative terminal states. Our model can be seen in terms of 5-HT's pruning of a decision tree of outcome states and choices [33,34].

We argue that the results on tryptophan depletion (TrD) above now emerge when considering the consequences of this reflexive behavioral inhibition on ongoing learning about the world, and on subsequent action choice and predictions. The most notable effect in the model is a critical *bias* toward optimistic valuation. That is, states and actions with potentially negative consequences are under-explored and incorrectly (over)-valued because of the reflexive inhibition. When inhibition fails, though, which is the last of the three issues mentioned above, there are two adverse consequences. First, the inhibition is no longer a crutch for instrumental action choice, so subjects have to learn to avoid potentially bad situations rather than being able to rely on this reflexive mechanism. Second, due to a mismatch between policy and value function, characteristic inconsistencies between the predicted and actual values arise, with the actual values encountered being more negative than predicted, though also actually more realistic. This mismatch between policy and value function also leads to an overall reduction in rewards obtained. Boosting 5-HT in the model again restores the status quo. Of course, this highly simplified model cannot possibly, by itself, accommodate all the diverse and confusing roles of 5-HT. Nevertheless, it replicates some prominent behavioral and pharmacological facets of depression and anxiety in humans and animal models, which we return to in the Discussion.

The next section defines the model of trains of thought more formally. The Results section considers normal (hence biased) learning, and the consequences of impairments to 5-HT processing. We save for the Discussion a broader discussion of data and theories pertaining to 5-HT.

## Methods

### The Model: Trains of Thought

**Figure 1:**
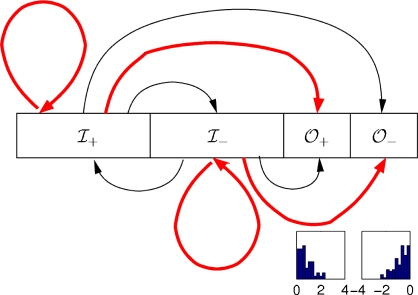
Markov Models of Thought The abstract state space is divided into the four blocks shown. The right two, *O℘*_+_ and *O℘*_−_, are associated with direct affective values *r*(*s*) (inset histograms); the left two, *I℘*_−_ and *I℘*_+_, are internal. Transitions between (belief) states are determined by actions (thoughts). We initially focus on a fixed policy, leading to the transition between states shown in the figure: states in each internal block *I℘*_+_ and *I℘*_−_ preferentially connect with each other and their respective outcome states *O℘*_+_ and *O℘*_−_. However, each state has links to states in the other block. The model is approximately balanced as a whole, with an equal number of positive and negative states.

Figure 1 illustrates our underlying model of trains of thought. It is intended to emphasize a role for 5-HT in behavioral inhibition, and is therefore couched at an abstract level. Throughout, we will equate thoughts with actions, and revisit the more general action setting later. We initially focus on the effect of one inhibitory reflexive action in the context of otherwise fixed actions (a fixed policy).

A train of thoughts starts at one of a set of internal belief states (*I℘*_+_, *I℘*_−_), may proceed through more such states, and ends in one of set of terminal outcome states (*O℘*_+_, *O℘*_−_). The connectivity between belief states is sparse, with states *I℘*_+_ leading preferentially to other states in *I℘*_+_ and outcome states *O℘*_+_ with positive values; and states *I℘*_−_ leading preferentially to other states in *I℘*_−_ and outcome states *O℘*_−_ with negative values (red arrows), though each could also lead to states of opposite “sign” (black arrows in Figure 1). In addition, trains of thought can be inhibited by 5-HT (see below). In this simple model, the value of an internal state is the average value of the terminal states to which it ultimately leads.

More formally, the model is a form of Markov decision process (see [35]), with four sets of sparsely interconnected states (*I℘*_±_, *O℘*_±_). Two sets, *O℘*_+_ and *O℘*_−_ (each with 100 elements in the simulation) are associated respectively with positive (*r*(*s*) ≥ 0, *s* ∈ *O℘*_+_) and negative affective values (*r*(*s*) ≤ 0, *s* ∈ *O℘*_−_); both are drawn from suitably truncated 0-mean, unit variance, Gaussian distributions (see inset histograms in Figure 1) and are terminal states. The other sets, *I℘*_+_ and *I℘*_−_ (each with 400 elements), contain internal states and are not associated with immediate affective values (*r*(*s*) = 0, ∀*s* ∈ *I℘*_−_ ∪ *I℘*_+_).

### Serotonergic Inhibition

A policy is a (probabilistic) mapping from states to actions *a* ← π(*s*) and defines the transition matrix between the states in the model. For simplicity, we consider a fixed, basic, policy *π* ^0^. In this, each element of *I℘*_+_ effectively has eight outgoing connections: three to other (randomly chosen) elements in *I℘*_+_; three to randomly chosen elements in *O℘*_+_; and one each to randomly chosen elements in *I℘*_−_ and *O℘*_−_. Similarly, each element of *I℘*_−_ has eight outgoing connections: three to other (randomly chosen) elements in *I℘*_−_; three to randomly chosen elements in *O℘*_−_; and one each to randomly chosen elements in *I℘*_+_ and *O℘*_+_. Thoughts are modelled as actions *a* following these connections, labelled by the identities of the states to which they lead. Text S1 gives details of a more complex environment in which we explicitly explore effects of impulsivity.

**Figure 2:**
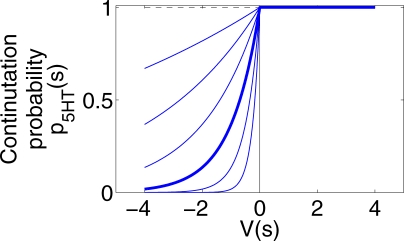
Probability of Continuing a Train of Thoughts For values *V*(*s*) > 0, thoughts are continued with probability 1. Conversely, when the state *s* has negative value, the probability of continuation drops of as an exponential function of the value. The rate of the exponential is set by *α*_5HT_.

To isolate the effect of 5-HT in inhibiting actions in aversive situations, we consider the highly simplified proposal that serotonin stochastically terminates trains of thoughts when these reach aversive states. More specifically, under serotonergic influence the transition probabilities are modified in a manner that depends on states' values. We let the probability of continuing a train of thought (of continuing along the fixed policy *π* ^0^) be dependent (and inversely related to) the value *V*(*s*) of a state:

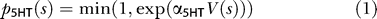
where *α*_5HT_ is a multiplicative factor that scales the impact of 5-HT (see Figure 2). When thoughts are not continued (inhibited), they stop and restart in a randomly chosen state *I℘* (though see below for relaxations of this). The more disastrous the potential sequelæ of state *s*, the more negative *V^π^*(*s*), and so the less likely the chain was to be continued. On the other hand, even slightly positive values would essentially veto any termination. This introduces an asymmetry into the model defined by the simple base policy. Other possibilities for the information reported by 5-HT and for the dynamic interaction between 5-HT and dopamine are considered in the discussion, and the fixed base policy *π* ^0^ is relaxed below.

### Learning

The value of each state represents the expected reward obtainable from that state when following a particular policy. Under the fixed policy *π* ^0^, dynamic programming techniques [35] allow the value function *V^π^*(*s*) over states *s* to be written, and solved for, concisely as: *V^π^*(*s*) = *r*(*s*), *s ∈ O℘_±_* , and

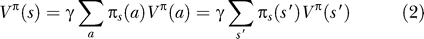
where *γ* is a discount factor (*γ* = 0.9 in our simulations). Dynamic programming also uses a Q^*^(*s*,α) function [36] over states and thoughts defined for those actions that exist by

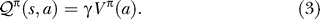



Optimal values *V* ^*^(*s*) and Q^*^(*s*,α) are those value functions associated with any policy 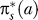 that maximizes the long-run affective outcomes of the train.

While it is not possible to use these techniques directly to evaluate the value function under serotonergic influence (the inhibition depends on the value function itself and thus represents a nonlinear interaction), the temporal difference learning rule [35] can be used to acquire estimates 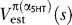 of the values 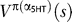 of states under serotonergically modified policies 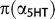 . The temporal difference learning rule specifies an online learning rule for which the change in the estimated value 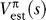 based on taking action α at state *s* and therefore arriving deterministically at *s′* = α(*s*) is:

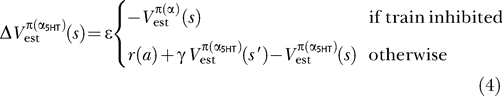
where the learning rate *ɛ* = 0.05. A slightly simpler alternative rule suggests that learning of 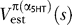 is itself prevented by termination:

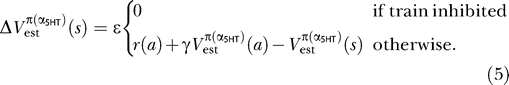



That 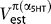 does not change under this rule given termination implies that learning is only slowed for these states, rather than being biased toward zero. We generally report results from this variant.

In the sequel, we show values after substantial learning (20,000 trains), plus the consequences of manipulating serotonin (by manipulating *α*_5HT_) once the values are already acquired.

### Manipulations After Learning

#### Tryptophan depletion.

Given the values 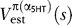 learned under a policy *π*(*α*_5HT_) determined by *α*_5HT_ = 20, the steady-state transitions probabilities can be calculated for any new *α*_5HT_ ≠ 20 simply by working out the probability of inhibition for each state. In particular, this allows *α*_5HT_ to be reduced to model a pharmacological or psychiatric reduction in serotonin function. To separate the effect of this reduction from that of learning, we only learn up to the reduction and then look at the behavior after the reduction in the absence of further learning.

#### Recall bias and reward seeking.

To account for the effect of recall biases often seen in depression, we will additionally consider the effect of biased resampling after behavioral inhibition. A simple way of achieving this in a manner that relates to the affective value of states is to let

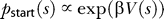
whereby values of *β* < 0 will bias resampling toward states with *lower* values *V*(*s*) (i.e., states in *I℘*_−_).

So far, only serotonergically determined inhibitory responses have been considered. Mirror to these are dopaminergically controlled approach responses [32], which actually favour actions α with positive state-action values *Q℘*(*s*,α) (under a policy). The combined effect can be incorporated in a straightforward manner by choosing action α in state *s* according to a softmax

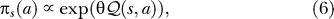
where *θ* controls the degree of influence of the *Q℘* value. Note that, in this simple model, instrumental and Pavlovian control are essentially indistinguishable.

## Results

### Behavioral Inhibition

**Figure 3:**
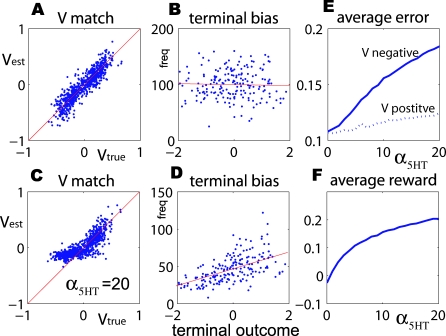
Learning with Behavioral Inhibition (A,B) With *α*_5HT_ = 0, for one particular learning run, the values *V*_est_ match their true values *V*_true_ (inferred through dynamic programming) under an equal-sampling exploration policy (A), and trains of thought end in terminal states *O℘*_−_, *O℘*_+_ equally often as a function of their actual outcomes (B) (the red line is the regression line). (C,D) With *α*_5HT_ = 20, negative *V* values are poorly estimated (since exploration is progressively inhibited for larger *α*_5HT_), and the more negative the value of the outcome, the less frequently that outcome gets visited over learning (D). Importantly, there is an optimistic *underestimate* of the negative value of state. (E) The root mean squared error (averaging over 20 runs) for states with positive (dotted) and negative *V*_true_ values as a function of *α*_5HT_. The effect of the sampling bias is strikingly apparent, preventing accurate estimates mainly of the negatively valued states. (F) Average reward received during learning as a function of *α*_5HT_—the benefits of behavioral inhibition are apparent.

By construction, the environment in Figure 1 is symmetric with respect to rewards and punishments, and so the overall statistics of the values of states are balanced about zero. Indeed, Figure 3A shows that for the base policy, 20,000 learning steps are ample to acquire a reasonable value *V*_est_(*s*) for the states (the remaining discrepancies from *V*_true_(*s*), here defined for *α*_5HT_ = 0, arise from the stochasticity in the choice of action together with the fixed learning rate). Critically, there is no bias in either *V*_est_(*s*) or *V*_true_(*s*).

By contrast, Figure 3D shows the substantial bias in 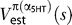 consequent on setting a large value of *α*_5HT_ = 20. In this case, low-valued states are much less well visited and explored. The bias comes despite the use of learning rule [5], which only slows down learning for low-value states rather than also distorting it. Of course, in this case, the extent of the bias depends on the initial values for the states (all of which are set to zero in the simulation).

Figure 3E shows how frequently each of the outcome states was reached in a run (as a function of its outcome *r*(*s*)). Since behavioral inhibition terminates trains on their way to potential disaster, aversive terminal states are sampled less (shown by the red regression line), which is consistent with the bias of the estimated value. Figures 3C and 3F show these effects as a function of *α*_5HT_. The greater the inhibition, the worse estimated the values are (Figure 3C), particularly for aversive states; however, the more benign is the exploration (Figure 3F). Learning with greater inhibition leads to a more optimistic set of values; however, this is coupled with a more aggressive rejection of all actions even mildly associated with negative outcomes.

### Tryptophan Depletion

Reducing the value of *α*_5HT_ after learning a value function 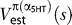 under its influence can be expected to have various consequences, as it introduces a mismatch between policy and value function. The most obvious one is a more negative average affective outcome (the average value of trains of thought) in the model. This is because choices are less biased against actions that are predicted to have aversive consequences, and so the latter occur more frequently. A second consequence is that there will be substantial adverse surprises associated with transitions that previously were inhibited. The surprise at reaching an actual outcome can be measured using the prediction error

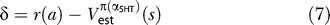
for the last transition of a chain from state 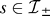 to a state 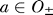 . We may expect negative prediction errors

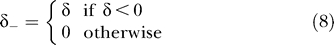
to be of special importance, because of substantial evidence that aversive outcomes whose magnitudes and timing are expected so they can be prepared for, have substantially less disutility than outcomes that are more aversive than expected (at least for physiological pains; see [37]).

**Figure 4:**
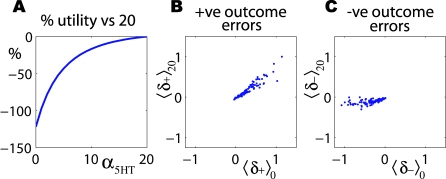
Reduced Inhibition These graphs show statistics of the effect of learning *V* values with *α*_5HT_ = 20, and then suffering from reduced serotonin *α*_5HT_ < 20 during sampling of thoughts. For a given thought environment, these are calculated in closed form, without estimation error. (A) As is also evident in Figure 3F, the average affective return is greatly reduced from the value with *α*_5HT_ = 20; in fact, for the extreme value of *α*_5HT_ = 0, it becomes slightly negative (reflecting a small sample bias in the particular collection of outcomes). (B,C) Normalized *outcome* prediction errors at the time of transition to *O℘*_+_ (B) or *O℘*_−_ (C) for *α*_5HT_ = 20 against *α*_5HT_ = 0. These reflect the individual probability that each terminal transition goes to *r*(*s*) from *V*(*s′*) for *s* ∈ *O℘* and *s′* ∈ *I℘*, including all the probabilistic contingencies of termination, etc. They are normalized for the two values of *α*_5HT_. Terminations in *O℘*_+_ are largely unaffected by the change in inhibition; terminations in *O℘*_−_ with negative consequences have greatly increased negative prediction error.

Figure 4 shows the consequences of learning under full inhibition and then wandering through state space with reduced inhibition. The change in the average terminal affective value as a percentage of the case during learning that *α*_5HT_ = 20 is shown in Figure 4A. As was already apparent in Figure 3F (which averages over the whole course of learning), large costs are incurred for large reductions in inhibition. For *α*_5HT_ = 0, the average reward is actually negative, which is why the curve dips below −100%. This value is relevant, since the internal environment is approximately symmetric in terms of the appetitive and aversive outcomes it affords. Subjects normally experience an *optimistic* or rosy view of it, by terminating any unfortunate trains of thought (indeed, 55% of their state occupancy is in *I℘*_+_ compared with *I℘*_−_). Under reduced 5-HT, subjects see it more the way it really is (the ratio becomes 50%).

Figure 4B and 4C show comparative scatter plots of the terminal prediction errors. Here, we consider just the last transition from an internal state to an outcome state. Prediction errors here that are large and negative, with substantially more aversive outcomes than expected, may be particularly damaging. Figure 4C compares the average terminal prediction errors for all transitions into states in *O℘*_−_ with no serotonergic inhibition *α*_5HT_ = 0, to those for the value *α*_5HT_ = 20 that were used during learning. For the case that *α*_5HT_ = 20, the negative prediction errors are on average very small (partly since the probability of receiving one is very low). With reduced inhibition, the errors become dramatically larger, potentially leading to enhanced global aversion. By comparison, as one might expect, the positive prediction errors resulting from transitions into *O℘*_+_ are not greatly affected by the inhibition (Figure 4B).

### Recall Bias

**Figure 5:**
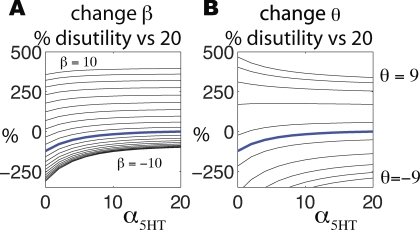
Reward Seeking and Recall Bias Both plots are in the same form as Figure 4A, showing the percentage utilities compared with the standard learning case *α*_5HT_ = 20, as a function of *α*_5HT_ (the emboldened blue curve is exactly that in Figure 4A). (A) Given a mood-dependent bias on the starting state, with 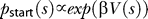 , the plots show the consequences of various values of *β*. Negative *β*, favoring low value states, leads to substantially negative average outcomes. (B) Instrumental control of action choice, a putative model of dopaminergic effects, can also either exacerbate or improve the outcomes, depending on the value of the parameter *θ* governing a softmax choice of actions.

Two additional effects enrich this partial picture. One, which plays a particularly important role in the cognitive behavioral therapy literature, is that depressed patients have a tendency to prefer to *recall* aversive states or memories [38,39]. Figure 5A shows the consequence of doing this according to a simple softmax (see Methods). These curves, as in Figure 4A, show the percentage average utility compared with *α*_5HT_ = 20, *β* = 0 across values of *α*_5HT_, and for *β* = −10, −9,…,10. As might be expected, biasing the starting point to *I℘*_−_, and, even worse, to those particular states in *I℘*_−_ that are most deleterious, has a big negative impact on average utility. For *α*_5HT_ = 0; *β* = −10, occupancy of *I℘*_+_ relative to *I℘*_−_ became a paltry 27% as subjects ruminate [40,41] negatively.

### Reward Seeking

The second factor is our restriction to just inhibition of trains of thought rather than a more fine-scale manipulation of the relative probabilities of different thoughts. We now relax this and explore the effect of additionally allowing preferential transitions toward certain states. In Equation 6, for positive values, the parameter *θ* biases action choice toward actions leading to positively valued states, whereas for negative values it does the opposite (i.e., subjects prefer to transition to negatively valued states). Figure 5B shows the effects of *θ*. It is apparent that rather extreme values of *θ* can both significantly aggravate or suppress the effect of *α*_5HT_. For the highest positive values of *θ* the curves reverse shape, showing that it can be beneficial *not* to inhibit trains of thought. This arises since the model of Figure 1 was chosen to have the extreme property that there is always the possibility of avoidance (in that all the states in *I℘*_−_ admit at least one action that leads to *I℘*_+_), and inhibiting trains of thought removes this outcome. A different, and rather counterintuitive, interaction between inhibition and reward seeking obtains in environments where rewards are hidden behind punishments (see  Text S1 and Figure S1).

## Discussion

We studied a very simple Markov decision process model of affectively charged thoughts, and showed various aspects of the influence of behavioral inhibition on the experience of appetitive and aversive outcomes, predictions, and prediction errors. The model formalises behavioral inhibition as a Pavlovian control process that arrests internally directed thoughts (and likewise externally directed actions) that are predicted to lead to aversive consequences. Overall this is favourable, leads to enhanced average rewards, and is related to adaptive pruning [33,34]. However, the consequences can also be deleterious [31,32]. Compromising inhibition in the model has two related consequences. First, the values of states are revealed to be overly optimistic. Second, control is disturbed, with aversive chains being insufficiently deselected.

While this work shows how several prominent aspects of serotonin's manifold putative functions and effects can be reconciled within a unifying framework, we acknowledge that we have neglected a wide range of other issues, and certainly do not claim that this is an exhaustive account of the data. There is also an interesting alternative view of 5-HT, such as that due to [42] who suggested that it is involved in controlling the appropriate timescale of behavior by determining the discount factor for future affective outcomes (parameter *γ* in Equation 2). In this theory, 5-HT depletion reduces the effective value of *γ*, making subjects appear more impulsive [43–45]. Our model captures impulsivity through reduced 5-HT more directly, suggesting that actions that are comparatively worse lose direct inhibition that was previously restraining them, and are therefore more likely to be executed.

### Behavioral Inhibition System

We suggested that this form of behavioral inhibition arises through predictions of aversive outcomes, tied to serotonin's putative role in reporting aversive prediction errors as an opponent to dopamine. This comes directly from the original notions of behavioral inhibition and serotonergic effects from Gray, Deakin, Graeff, and their colleagues [6,7,13,29,30]; however, it is perhaps best seen as a subset of the current version of Gray's BIS [29]. One salient difference is that BIS is suggested as being primarily engaged by *conflict*, rather than ongoing predictions of future aversive outcomes. Of course, a main source of conflict is that between approach and avoidance, with the latter coming from these aversive predictions. An interesting consequence of dividing the prediction of the value of future outcomes between two separate opponent systems is that it is indeed possible to have simultaneous appetitive and aversive expectations, as opposed to just one combined net prediction. Although we used the net prediction to control inhibition, it would be interesting to explore other possibilities associated with the BIS view, such as that any aversive prediction could arrest ongoing action, even if outweighed by appetitive predictions.

Further, rather than have the aversive predictive *values* of states lead to termination of trains of thought, it is possible that the negative prediction error (*δ*_−_ from Equation 8), which Daw et al. [10] suggested is being reported by phasic serotonin, could be responsible instead. Alternatively, in the mirror reflection of the proposal that a tonic dopaminergic signal reports average reward (and controllable/avoidable punishment) and energises behavior [46,47], it could be that a more tonic serotonergic signal, averaging aversion over longer time horizons and favoring quiescence, could be responsible.

Another difference between our account and the full BIS is that, in the latter, although actions are indeed inhibited in the face of conflict, the BIS is then suggested as initiating a set of behaviors (such as exploration or risk assessment) to resolve that conflict. The set of preparatory Pavlovian actions associated with aversive predictions appears to be more refined than that associated with appetitive predictions (mostly just approach), with a wide range of different defensive possibilities being selected between according to the nature and proximity of the threat [30,48]. One class of these is even laid out along columns of the dorsal periacqueductal gray (PAG [49]). Nevertheless, any of these defensive manoeuvres would interrupt the ongoing chain of actions, and this is what we modelled. Risk assessment and exploration are of most obvious use in the face of uncertainty and ignorance, whereas conditioned suppression, and thus the sort of inhibition that we consider, remains even after substantial learning. It would certainly be worth going one stage further, modelling the interruption in terms of a switch between different Markov decision problems, with new information changing the transition and payoff structures.

### Tryptophan Depletion

One of our central results is the effect of an acute reduction in *α*_5HT_ *after* learning with elevated *α*_5HT_ has taken place. In our model, this leads to a *decrease* in behavioral inhibition of actions leading to negative states. Although specific effects might arise from local manipulations of 5-HT concentrations or receptor responsivity, key data come from the systemic manipulation associated with acute TrD [50], in which plasma levels of tryptophan and, at least in animals, central nervous system levels of serotonin, are drastically reduced (by up to 90%). Although the particular chains of thoughts analysed here have not been the subject of experimental scrutiny, there is by now a considerable body of literature on the effects of TrD on normal human functioning. In broad agreement with our results, various effects have been related to decreased reward processing [39,51,52], decreased behavioral inhibition [44,53–57], rumination [21], facial fear recognition [58], and, more indirectly, increased aggressiveness [54,59,60].

Perhaps of most direct relevance to our implementation are the results of a recent study which decoupled rewards from correct performance of an action from the outcomes of the actions [61]. This study actually involved a sophisticated assessment of the effects of TrD on reversal learning. However, one way of viewing a portion of the results stems from an abstract representation of the task. Subjects had to press one of two buttons (A or B) in response to one of two stimuli (also called A and B), with presses associated with A leading to a symbolic reward and presses associated with B leading to a symbolic punishment. Critically, these outcomes were independent of the rectitude of the subjects' responses, so they couldn't avoid the punishment by making errors. In this case, subjects more often failed to press button B correctly than button A, and this difference disappeared after TrD. This is directly consistent with the present interpretation of serotoninergic inhibition of actions that lead to aversive outcomes.

Famously, TrD does not have a uniform effect on all subjects. There is an important genetic polymorphism in the 5-HT reuptake mechanism, with subjects having the less efficient version generally showing greater effects [52,57,62–66]. For this to be consistent with our formulation, the difference in functional 5-HT levels before and after TrD has to be greater in the subjects with less efficient reuptake. This in turn might most simply be due to increased levels of 5-HT (and behavioral inhibition) throughout development in carriers of the short 5HTTLPR allele. Perhaps related to this is the finding that TrD produces a dose-dependent relapse of depressive symptomatology in formerly depressed patients [18–20,41], or in patients with risk factors such as a family history of depression [63] (although the three-way interaction between TrD, 5HTTLPR, and past depression is hard to fit into this framework [67]).

There is a significant body of work on the effects of serotonergic manipulations on affective processing, particularly on processing of facial expressions [58,68–70]. It is difficult to interpret this work in our context for several reasons: first, there have often been effects on recognition of specific aversive facial expressions (e.g., fear) but not others (e.g., disgust). Our model does not speak to these distinctions. Second, in these tasks, subjects identify stimuli by pressing a button. Thus, there is a Pavlovian association between certain buttons and the aversive stimuli, and, interpreting these tasks in the same framework as we interpreted the work of Cools et al. [61], one might predict that TrD would increase rather than decrease accuracy. The precise effect, however, would depend on the relative strength of the instructed and the reflexive Pavlovian response, and on the antagonism between the responses. Indeed, both aspects have been found: acute manipulation of serotonin increased recognition accuracy of fearful faces with increasing serotonin [58,68,70], whereas a more chronic increase in 5-HT (via SSRIs) yields a decrease in recollection of negative memories [69]. Furthermore, while the exact relationship between behavioral inhibition and amygdala activation still needs clarification, it is additionally possible that increased amygdala activation may relate to perceptual mood congruency effects [38]: after disinhibition, thoughts often visit negative states, and it is possible that this may affect prior expectations about stimulus which in turn could speed up processing of negatively valenced information.

TrD (or indeed SSRIs) have not previously been used in tasks like the Markov decision problem of the type we discussed. A direct prediction of the model is that subjects trained under TrD would explore states less when tested in a normal regime, while those trained under SSRIs would do so more (assuming that SSRIs indeed elevate 5-HT levels). Similar predictions hold for subjects with short or long alleles of the 5-HT reuptake mechanism on these tasks. This would essentially represent a generalisation of the findings by Cools et al. [61] to the domain of sequential decisions. The tasks could use external, observable actions; more directly, it would also be useful to monitor the execution of affective trains of thought, and study the perturbation of this under serotonergic manipulations. In designing such studies, it is important to bear in mind the potentially opponent instrumental and Pavlovian effects, in just the same way that boosting dopamine and monitoring the effects on negative automaintenance may be confusing. Note that although there are various important datasets as to the effects of TrD on simple probabilistic and delay-discounting tasks [51,52,56,71–75], these studies do not encompass the sorts of behavioral chains that we propose 5-HT to be able to halt.

### Dopamine and Serotonin

One of the backdrops for the present theory was the extensive modeling of phasic dopamine as a prediction error for future reward, and the results that (1) the baseline firing rates of dopamine cells are insufficient to report prediction errors for negative rewards (i.e., punishments); (2) the ample psychological evidence for the existence of a pair of systems, one associated with appetitive outcomes and the other with aversive outcomes; and (3) the evidence that at least some aspects of 5-HT and dopamine are in mutual opposition. Indeed, based on these data and the theories of Deakin and Graeff [6,7], Daw et al. [10] suggested that serotonin rather than dopamine reports negative prediction errors based on an antagonism between serotonin and dopamine at both a behavioral and pharmacological level. For example, in rodents, 5-HT antagonises the general excitatory effects of dopamine [4], the self-administration of amphetamine and intracranial self-stimulation [76,77], the effects of dopamine on appetitive learning [8], and the potentiation of appetitive learning by amphetamine [78].

However, pure opponency is far too simple. For instance, there is by now extensive evidence that 5-HT modulates dopaminergic activity both through receptors in the ventral tegmental area and by modulation of distal release sites, and that this modulation can occur in both inhibitory and excitatory directions [4,77,79–89]. Even the rise in 5-HT due to SSRIs has overall pro-dopaminergic effects, both at behavioral and physiological levels [81,90–92], and there is one report that DA antagonists reverse the antidepressant effect of SSRIs [93]. Further, there is evidence that DA itself is released in many aversive circumstances [94,95], and is involved in aversively motivated behaviors like avoidance [96–98].

In our terms, apart from the aspects of the interaction of dopamine and 5-HT that were explored in Figures 5B and S2, there are a couple of other effects. First, inhibition in our model has the consequence of increasing the average expected reward. As such, tonic dopamine, which has been suggested to report such a quantity [46,47,99–101], would be increased when 5-HT is boosted, and potentially vice versa [88,92]. This would compete with the more direct effect that 5-HT inhibits actions, and particularly inhibits actions supported by dopaminergic predictions or rewards [8,78,102], and thus high levels of 5-HT might also depress levels of tonic dopamine, more in line with accounts that stress the opponent role of dopamine and serotonin [4,10,103].

The second complexity (Boureau, personal communication) is that active defense (such as active avoidance) requires energizing, and indeed appears to be controlled by the (presumably dopaminergically reported) appetitive outcome of reaching a state of safety rather than the (presumably serotonergically mediated) outcome of leaving a state of fear. That is, it appears that dopamine reports the rewards reaped from avoiding or controlling aversive outcomes [15,94,104].

We mentioned the mirror notion that the relationship between 5-HT and inhibition arising through aversive predictions is parallel to the obverse relationship for dopamine and engagement/approach through appetitive predictions [32]. In this case, appetitively directed chains of thoughts would be favored. Indeed, Smith et al. [105,106], in their work on the conditioned avoidance model of schizophrenia, suggested something rather like this. In their account, dopamine controls the extent of search through a forward model, although they did not couple this to dopamine's involvement in appetitive prediction.

In all, disentangling and elucidating these varied relations between dopamine and serotonin is a pressing task.

### Depression and Anxiety

It would be reasonable to argue that the present model is more relevant to anxiety than depression. There is at best a somewhat fuzzy distinction between the two in terms of risk factors [107] and pharmacology [108], and they are extraordinarily co-morbid [109]. There is also no complete definition of either disease in terms of the sort of reinforcement mechanisms that we have been considering.

While depressive (but not anxious) symptoms can be reinduced by TrD in a subpopulation of patients, TrD it is not the only such manipulation, and it is not effective in all patients. Patients who are responsive to seratonin–norepinephrine reuptake inhibitors (SNRIs) are more sensitive to catecholamine depletion by *α*-methyl-tyrosine [110,111] than TrD, and a recent report with a DA antagonist successfully re-induced depressive symptoms in formerly depressed people [93]. The latter authors suggest that DA may be a “final common path” for depression, and may relate more to the depressive state than serotonin, which in turn may be more important in defining a trait [15,112,113]. In addition, only 50% of formerly depressed subjects do respond to TrD [41,114], and a pooled analysis of 71 formerly depressed subjects found that previous response to SSRIs had less predictive power for TrD response than chronicity of the depressive disorder and sex [114]. As mentioned, resolving the actual relative contribution of serotoninergic inhibition will be tricky.

### Conclusions

In sum, the findings in this study argue for an involvement of the serotonin reuptake mechanism in mood disorders such as anxiety and depression in the following manner: due to a decreased efficiency of the transporter, increased behavioral inhibition results in acquisition of overly optimistic values. Such value functions are adaptive, but only in conjunction with strong behavioral inhibition. On the other hand, they do render the individual highly sensitive to large decreases in average experienced rewards when serotonin function is reduced. This might underlie a (controversially) larger sensitivity to TrD and SSRIs of persons with the short 5HTTLPR allele (see [115]). Returning to the sequential decision-making tasks suggested above, this study would predict that the short 5HTTLPR allele would be associated with more reflexive avoidance of states predictive of punishment, and it may be possible to assess this with differential effects of TrD on carriers with the short and long 5HTTLPR allele.

A further, more involved conjecture, which returns to the fact that serotonin is not the sole causative agent in depression, is that it is the effects of reduced 5-HT on affective experience that leads to the various symptoms of depression, acting via the otherwise normative operation of the multiple systems involved in behavioral control. For instance, we have argued that the consequences of 5-HT reduction include unexpected punishments, large negative prediction errors, and a drop in average reward. These changes in the statistics of reward demand explanation, for example in terms of a shift in the characteristics of the environment, and should cause *normative* behavioral responses. In particular, the unsignalled aversion that comes independent of the subject's actions can be seen as a form of uncontrollable punishment. Uncontrollability lies at the heart of an important characterization of depression centred around learned helplessness [104,116].

We concentrated on the effects of reduced 5-HT rather than on the reasons for this reduction. The obvious option is that it is a pathological result from processes operating at a purely cellular level. However, it could also arise as a normative meta-adaptation to the statistics of experienced punishments and rewards. Formalizing this fully would require a more general theory of inhibition—what level of inhibition is optimal? Tools for the characterisation of the trade-off between accurate knowledge about a state's value and the cost incurred in learning about it are already in existence [34,117,118] and might be applicable to aspects of the present case.

## Supporting Information

Figure S1A Deep EnvironmentSimilar state space to Figure 1, but with a more explicitly deep structure. State in 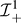 mainly lead to 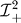 , or back to themselves. The last states in each of the two chains (here 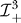 and 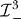 ) always preferentially lead to the outcome state *O℘*_+_ and *O℘*_−_.
(32 KB PDF)

Figure S2Inhibition in a Deep EnvironmentThe outcomes *O℘* are approached by sequentially walking through *K* = 4 levels. Only *I℘* ^4^ states lead to outcomes.(A,D) True values without inhibition are shown by the black line. It is constant for each level and valence, or illustration, as all outcomes were assigned the same positive value (+1 or −1). The reward of the states *I℘* is zero and shown by the dash-dotted line. The grey point display the estimated values of the states under inhibition *α*_5HT_ = 20. There is a positive bias in all states, but it is more pronounced in the states with true negative values. In (D), the dash-dotted line indicates that states 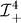 now carry reward −0.4, while states 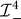 carry reward +0.4. States 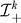 for *k* = (1,2,3} now have true negative values, and 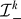 for *k* = (1,2,3} have true positive values.
(B,E) Probabilities of ending thought sequence in *O℘*_+_ or *O℘*_−_.(C,F) Effect of preferentially choosing actions according to their valence on the average value of states. The arrow indicates increasing *γ*. In (C), larger *γ* are advantageous, in (F), smaller *γ* are better.(210 KB PDF)

Text S1 Impulsivity in a Deep Environment (34 KB PDF)

## Abbreviations

BIS: behavioral inhibition system
SSRI: selective serotonin reuptake inhibitor
TrD: tryptophan depletion
