# Supplementary material for: Serotonin, Inhibition, and Negative Mood
Source: PLoS Comput Biol. 2008 Feb 1;4(2):e4. doi: 10.1371/journal.pcbi.0040004 (PMC2222921; doi:10.1371/journal.pcbi.0040004)
Supplement: Supplementary file 1 [file pcbi.0040004.sd001.pdf]

Text S1 Impulsivity in a deep environment.

Supplementary Text S1 for Dayan P, Huys QJM (2008) Serotonin, inhibition and negative mood. PLoS Comput Biol 4(1):e4 doi:10.1371/journal.pcbi.0040004.sd001

The present implementation of inhibition could lead to short-sighted actions in deeper environments. To explore this, we consider an environment with explicit deep structure, where all large rewards are hidden behind small punishments, and vice-versa. Large $\alpha_{5\text{HT}}$ may prevent visits to the states associated with small punishments and thus prevent visits to the states behind them that are associated with large rewards. This effect is closely related to impulsivity, which itself has been associated with an impaired behavioural inhibitory system [55]. We first show that all the conclusions up to now also apply to an environment of depth K in which the reward structure is still bipartite, i.e. in which no positively valued states are primarily accessible via negatively valued states.

Supplementary Figure 1 shows the slightly augmented environment that we used to explore the effects of impulsivity. In this, the sets of non-terminal states are subdivided into K groups (Supplementary Figure 1 shows this for $K = 3$). The last of the groups ($\mathcal{I}_+^K$ is connected to the terminal states $\mathcal{O}_-$, as before; for the other groups with $k < K$, the role of $\mathcal{O}_+$ is replaced by $\mathcal{I}_+^{k+1}$. The same applies to the states with negative valence. Identical probabilities as for Figure 1A are used.

Specifically, we let states $\mathcal{I}_+^k$ of positive valence and at level $1 \leq k \leq K-1$ preferentially lead to states in the same level and valence $\mathcal{I}_+^k$ (with probability 3/8) or to states $\mathcal{I}_+^{k+1}$ (with probability 3/8) of the same valence, but one level closer to the outcomes $\mathcal{O}$. With smaller probability (1/8) they can also lead to states of the opposite valence at the same level $\mathcal{I}_-^k$ or one level up $\mathcal{I}_-^{k+1}$. States $\mathcal{I}^{K-1}$ have connections as shown in Figure 1B and are the only states that can lead to outcomes $\mathcal{O}$.

Supplementary Figure 2A shows the true values of states without inhibition and their estimated values with inhibition. There is a clear positive bias for all negatively valued states. Supplementary Figure 2B shows that the outcomes are still more frequently positive than negative, and Supplementary Figure 2C shows the effect of altering θ. As in Figure 5B, negative θ lead to preferential selection of actions leading to states with negative values, and the overall average value is increased by increasing θ.

However, the situation changes if the states with large positive outcomes are primarily accessible through punished states. The dash-dotted line in Supplementary Figure 2D shows that the states $\mathcal{I}_+^K$ at the final level K before the outcomes are now punished, while the states $\mathcal{I}_-^K$ are rewarded. The values of states $\mathcal{I}_+^3$ is now more negative than their counterpart without inhibition. This is because thoughts are often interrupted before they can proceed through (the punished) $\mathcal{I}_+^4$ to (the rewarded) $\mathcal{O}_+$. Overall, the states in $\mathcal{I}_+$ are now predominantly negative, and those in $\mathcal{I}_-$ predominantly positive. However, inhibition does still lead to an overall positive outcome bias (Supplementary Figure 2E), and diminishing $\alpha_{5\text{HT}}$ with $\theta = 0$ is still unfavourable (Supplementary Figure 2F, black line). However, the effect of θ is now reversed (see arrow in Supplementary Figure 2F): Negative θ (less reward seeking) will now predominantly lead to choices in $\mathcal{I}_+$, and thus to choices with longer-term positive outcomes (as there is no more inhibition). Thus, when $\alpha_{5\text{HT}}$ is lowered in an environment in which rewards lurk behind punishments, a reduction of reward-seeking behaviour may play a role in adaptively compensating for the lack of inhibition.
