# Supplementary material for: Serotonin, Inhibition, and Negative Mood
Source: PLoS Comput Biol. 2008 Feb 1;4(2):e4. doi: 10.1371/journal.pcbi.0040004 (PMC2222921; doi:10.1371/journal.pcbi.0040004)
Supplement: Supplementary file 2 [file pcbi.0040004.sg001.pdf]

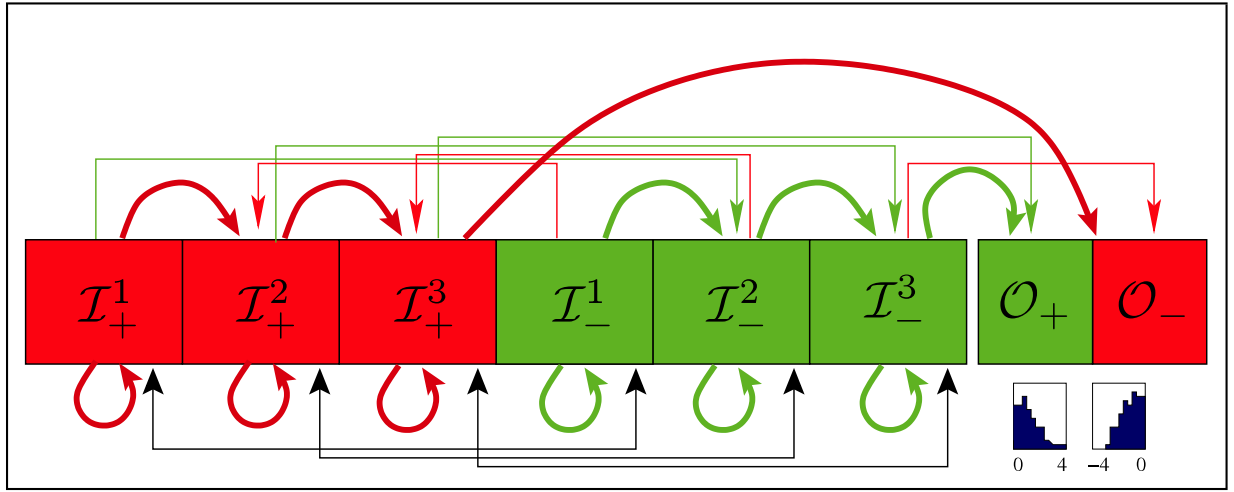

Supplementary Figure 1: Similar state space to Figure 1, but with a more explicitly deep structure. State in $\mathcal{I}_+^1$ mainly lead to $\mathcal{I}_+^2$, or back to themselves. The last states in each of the two chains (here $\mathcal{I}_+^3$ and $\mathcal{I}_-^3$) always preferentially lead to the outcome state $\mathcal{O}_+$ and $\mathcal{O}_-$.

Supplementary Figure 1 for Dayan P, Huys QJM (2008) Serotonin, inhibition and negative mood. PLoS Comput Biol 4(1):e4 doi:10.1371/journal.pcbi.0040004.sg001
