# Supplementary material for: Serotonin, Inhibition, and Negative Mood
Source: PLoS Comput Biol. 2008 Feb 1;4(2):e4. doi: 10.1371/journal.pcbi.0040004 (PMC2222921; doi:10.1371/journal.pcbi.0040004)
Supplement: Supplementary file 3 [file pcbi.0040004.sg002.pdf]

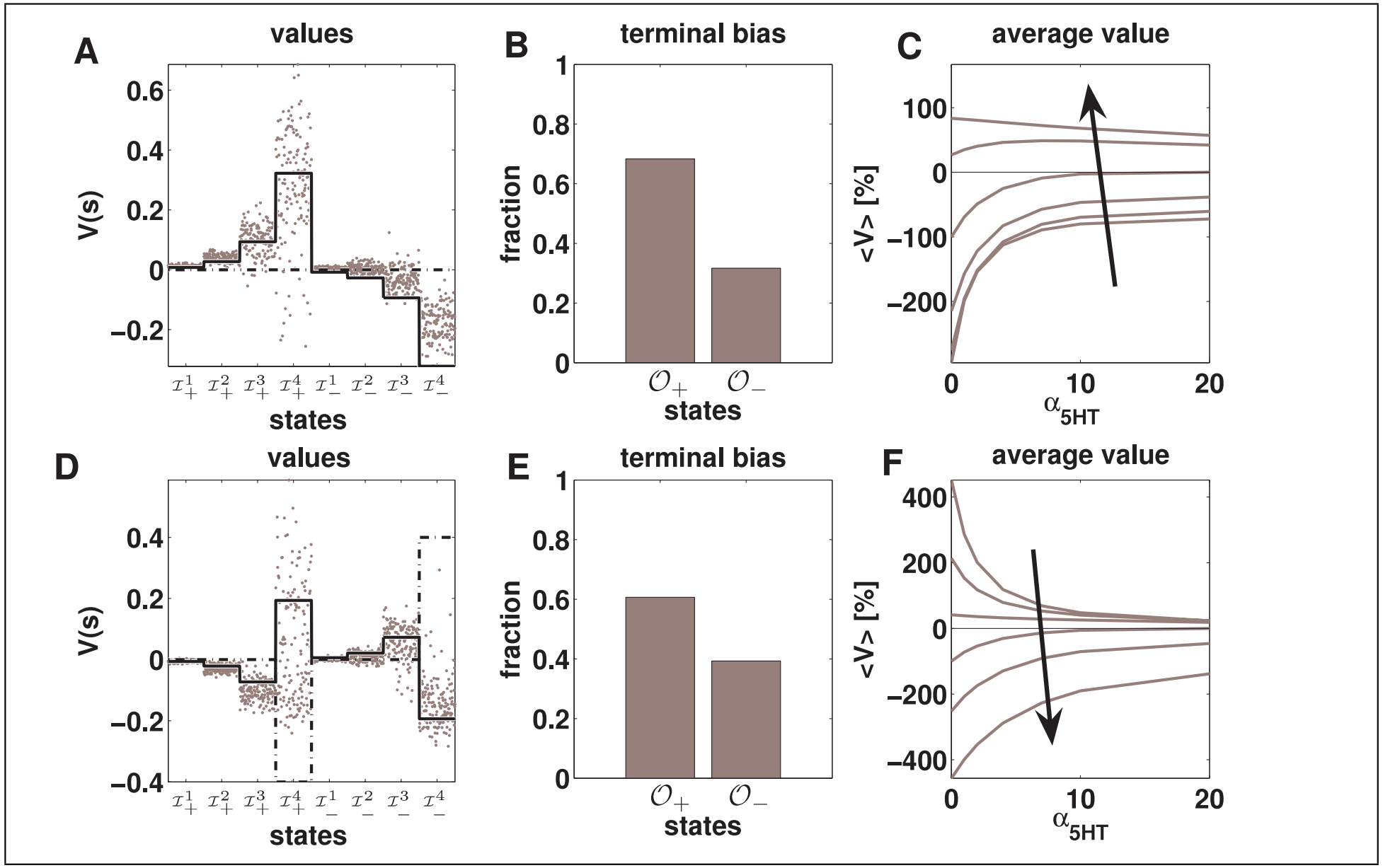


Supplementary Figure 2: Inhibition in a deep environment. The outcomes $\mathcal{O}$ are approached by sequentially walking through $K = 4$ levels. Only $\mathcal{I}^4$ states lead to outcomes. (A,D): True values without inhibition are shown by black line. It is constant for each level and valence as, or illustration, all outcomes were assigned the same positive value (+1 or -1). The reward of the states $\mathcal{I}$ is zero and shown by the dash-dotted line. The grey point display the estimated values of the states under inhibition $\alpha_{5HT} = 20$. There is a positive bias in all states, but it is more pronounced in the states with true negative values. In (D), the dash-dotted line indicates that states $\mathcal{I}^4_+$ now carry reward $-0.4$, while states $\mathcal{I}^4_-$ carry reward $+0.4$. States $\mathcal{I}^k_+$ for $k = \{1, 2, 3\}$ now have true negative values and $\mathcal{I}^k_-$ for $k = \{1, 2, 3\}$ have true positive values. (B,E): Probabilities of ending thought sequence in $\mathcal{O}_+$ or $\mathcal{O}_-$. (C,F): Effect of preferentially choosing actions according to their valence on the average value of states. The arrow indicates increasing $\theta$. In (C), larger $\theta$ are advantageous, in (F), smaller $\theta$ are better.

Supplementary Figure 2 for Dayan P, Huys QJM (2008) Serotonin, inhibition and negative mood. PLoS Comput Biol 4(1):e4
doi:10.1371/journal.pcbi.0040004.sg002
